# Sustaining planetary health through systems thinking: Public health's critical role

**DOI:** 10.1016/j.ssmph.2021.100844

**Published:** 2021-06-11

**Authors:** Hari S. Iyer, Nicole V. DeVille, Olivia Stoddard, Jennifer Cole, Samuel S. Myers, Huichu Li, Elise G. Elliott, Marcia P. Jimenez, Peter James, Christopher D. Golden

**Affiliations:** a Division of Population Sciences, Dana-Farber Cancer Institute, Boston, USA; b Department of Epidemiology, Harvard T. H. Chan School of Public Health, Boston, USA; c Channing Division of Network Medicine, Brigham and Women's Hospital, Boston, USA; d Department of Nutrition, Harvard T. H. Chan School of Public Health, Boston, USA; e Geography Department, Royal Holloway University of London and Royal United Services Institute, London, United Kingdom; f Department of Environmental Health, Harvard T. H. Chan School of Public Health, Boston, USA; g Division of Chronic Disease Research Across the Lifecourse, Department of Population Medicine, Harvard Medical School and Harvard Pilgrim Health Care Institute, Boston, USA

**Keywords:** Environmental health, Planetary health, Nutrition, Cities, Air pollution, Physical activity, Public health

## Abstract

Understanding and responding to adverse human health impacts of global environmental change will be a major priority of 21st century public health professionals. The emerging field of planetary health aims to face this challenge by studying and promoting policies that protect the health of humans and of the Earth's natural systems that support them. Public health, drawing on its experience of guiding policies to improve population health, has contributed to planetary health's development. Yet, few public health practitioners are familiar with planetary health's systems-oriented approaches for understanding relationships between economic development, environmental degradation, and human health. In this narrative review, we present key planetary health concepts and show how systems thinking has guided its development. We discuss historical approaches to studying impacts of economic development on human health and the environment. We then review novel conceptual frameworks adopted by planetary health scientists to study and forecast impacts of policies that influence human health and Earth's natural systems at varying spatiotemporal scales. We conclude by presenting examples of how applying the “Doughnut” model (an economic framework where the needs of people are met without overshooting the world's ecological limits) could guide policies for promoting health co-benefits to humans and natural systems.

## 1 Introduction

A failure to account for the long-term impacts of human activities on our planet's natural systems has caused shocks to societies' health and our ability to deliver health care. A global pandemic of the novel SARS-COV-2 virus, the like of which had long been predicted as a consequence of changing land use patterns, increased urbanization, natural evolution, climate changes and human encroachment in previously undisturbed habitats (Daszak, Cunningham, & Hyatt, 2000; Garrett, 1995; Gibb et al., 2020; MacKenzie, 2017; Plowright et al., 2017), has claimed over three million lives worldwide (Dong, Du, & Gardner, 2020). Dramatic increases in wildfires and hurricanes in the last 20 years, mediated by climate change, have killed many and destroyed livelihoods around the world (Marlon et al., 2012; Raymond et al., 2020). In addition, poorer mental health (Usher et al., 2020), “ecological grief” from anticipated loss of loved species and ecosystems (Cunsolo & Ellis, 2018), undernutrition (Akseer, Kandru, Keats, & Bhutta, 2020), and disruption to timely detection and treatment for chronic diseases (Einstein et al., 2021; Richards, Anderson, Carter, Ebert, & Mossialos, 2020; Wadhera et al., 2021) have increased as a result of pandemic and climate-related disasters (Greenough et al., 2001). As privileged population groups buffer themselves from the adverse health impacts of these crises through material and societal advantages, the burden has fallen on vulnerable and marginalized members of our societies (Bassett, Chen, & Krieger, 2020; Hotez, Bottazzi, Singh, Brindley, & Kamhawi, 2020; Smith and Ezzati, 2005). Eventually, all of humanity will be unable to avoid these threats – as demonstrated by the increasing severity of weather-related disasters in both low- and high-income countries (Ummenhofer and Meehl, 2017).

Faced with growing health and environmental challenges, public health has much to offer. The foundational knowledge of public health draws from numerous academic disciplines, from biomedical to social sciences. Many of the successes in public health – from the global HIV response to tobacco control – arose from the field's long history of using evidence-based science to identify threats to human health, and then successfully working with diverse stakeholders to translate that evidence into policy (Emmons, Kawachi, & Barclay, 1997; Farmer, Kim, Kleinman, & Basilico, 2013; Shilts, 2000). Public health studies identifying associations between increased ultraviolet radiation and skin and eye disease incidence informed the international Montreal Protocol to protect the stratospheric ozone layer by reducing production and consumption of ozone-depleting substances (Norval et al., 2011; UNEP Division of Technology, Industry and Economics OzonAction Programme, 2015).

Though not explicitly described as such, systems thinking has often underpinned public health's greatest achievements, by embracing the complexity in causal pathways, social structures, and policy actors that govern health (Leischow & Milstein, 2006). Four key concepts underpin systems thinking (Box 1): distinctions (what the problem is and is not), systems (part-whole relationships between problems and their antecedents), perspectives (whose views are brought to bear on the problem), and relationships (types and degrees of associations between problems) (Cabrera & Cabrera, 2016). Within the systems thinking framework, public health practitioners often focus on distinctions (categorizing populations, exposures, diseases) and relationships (identifying causal effects of individual exposure-disease pairs). Traditional public health research has historically been guided by theories of causation limited to biophysical phenomena and interventions delivered within the health care system, reflecting a reductionist, rather than systems-oriented, conceptual framework (Krieger, 2011a, pp. 126–152). This perspective, which served as dogma (Engel, 1977) for public health scientists and major funding agencies in the United States through the latter half of the twentieth century (Harden & Hannaway, 2001), often minimized theories that socio-ecological processes, rather than biomedical processes, influence disease patterns at population level (Krieger, 2011a, pp. 126–152). In this paradigm, multilevel systems are nuisance parameters to be adjusted away instead of context that can inform implications of study findings (Krieger, 2011b, pp. 202–235). Failure to explicitly consider systems and diverse perspectives when interpreting and presenting findings may prevent a more wholistic understanding of causes of disease and consideration of multilevel impacts of policies (Krieger, 2011b, pp. 202–235; Krieger & Davey Smith, 2016; McMichael, 1999; Midgley, 2006). Explicit consideration of perspectives and systems domains would ensure that public health recommendations consider diverse scientific evidence when advocating for policies and consider consequences of policies at multiple human and planetary scales to adequately account for benefits and harms (Krieger, 2011b, pp. 202–235; McMichael, 2017; Myers & Frumkin, 2020; Redvers, 2021; Robinson et al., 2018).

Box 1 **Glossary**

**Table:** 

**The Doughnut**: Economic model proposed by Raworth that frames safe operating space for humanity as lying within planetary boundaries specifying environmental thresholds that should not be transgressed, whilst achieving twelve social standards that should be reached to optimize human thriving (Raworth, 2017). **Earth system**: Encompasses Earth's interacting physical, chemical, and biological processes. Focuses on physical properties of the entire globe (land, oceans, atmosphere and poles) and geochemical cycles (for example, carbon, water, and nitrogen cycles). **Ecosystem**: A biological system composed of all the organisms found in a particular physical environment, interacting with it and with each other (OED Online, n.d.). **Gaia Theory**: Unifying model proposed by Lovelock for understanding how living organisms and their physical environments interact in synergistic ways to maintain climate and biochemical conditions that sustain life. The global ecosystem, understood to function in the manner of a vast self-regulating organism, in the context of which all living things collectively define and maintain the conditions conducive to life on earth (Lovelock & Margulis, 1974). **Great Acceleration**: Term proposed by Steffen to describe the rapid increases in human population and activities and accompanied by rapid changes to climate, geochemical, water, land, and ecosystems in the latter half of the twentieth century (Steffen et al., 2004). **Natural systems**: Systems that occur in nature (for example, water cycle through rain, ground water, evaporation, and evapotranspiration by plants, climate, weather and atmosphere, nutrient cycles through the food chain) **Panarchy**: A theory to explain human and natural systems behavior through cycles of four state transitions which, in response to external and internal shocks, may prompt either adaptation of the system to maintain its equilibrium, or transformation of the system into a new one with a different equilibrium. The four states of the cycle are (1) exploitation, (2) conservation, (3) release, and (4) reorganization. The exploitation and conservation phases are characterized by stability, while the release and reorganization phases are characterized by responses to shocks and more prone to transitions into new equilibria. At each state, the system exhibits different levels of resilience (ability to maintain stability in face of shocks), potential (accumulated resources), and connectedness (how strong system components are linked in face of a shock). (Gunderson & Holling, 2002) **Planetary Health**: field of study that aims to produce knowledge regarding relationships between human activities, their impacts on environment and downstream consequences to health of humans, other living organisms, and natural systems (Myers & Frumkin, 2020). **Planetary Boundaries**: A set of nine thresholds for Earth systems (air pollution, ozone layer depletion, climate change, ocean acidification, chemical pollution, nitrogen and phosphorus loading, freshwater withdrawals, land conversion and biodiversity loss) identified by Rockström and colleagues that must not be transgressed in order to preserve human life (Rockström et al., 2009). **Social-Ecological Systems:** term that describes idea that humans are part of nature, rather than separate from it. Human social systems and natural systems interact in dynamic, non-linear ways (exert feedback loops). These interactions can produce resilience in the system, in which its states are preserved even in response to external shocks, or they can reduce resilience of the overall system, which can lead to adverse consequences for humans and their environment. Importantly, these interactions operate at varying spatiotemporal and organizational scales (Ostrom, 2009). **Systems thinking:** Conceptual framework that organizes the understanding of complex systems through four rules: organizing ideas by distinctions, systems, relationships, and perspectives. In this framework, distinctions refer to how elements in a system have identities and can be grouped by what they are not, systems refer to elements that may be parts or a whole, relationships refers to associations between elements and their causal ordering, and perspectives refers to the viewpoint from which elements are analysed (Cabrera & Cabrera, 2020).

Alt-text: Box 1

In this narrative review, we present key concepts from the emerging academic field of planetary health and describe how it integrates systems thinking principles to guide policies that offer co-benefits to human health and the health of natural systems. We show how failure to apply systems-wide perspectives when considering impacts of economic development have, in the past, led to misguided policy prescriptions for how to prioritize economic development, health, and the health of natural systems. We discuss recent frameworks that incorporate systems-based conceptual models and demonstrate how they have identified sustainable planetary health policies that offer co-benefits to human health and the health of natural systems. A glossary (Box 1) is provided with definitions of key terms used throughout the article.

### 1.1 A brief overview of planetary health

Planetary health is defined as “the achievement of the highest attainable standard of health, wellbeing, and equity worldwide through judicious attention to human systems—political, economic, and social—that shape the future of humanity and the Earth's natural systems that define the safe environmental limits within which humanity can flourish” (Whitmee et al., 2015). This formulation articulates a systems-level conceptual framework for understanding the relationships between the health of humans and natural systems. Like ecosocial theories of causation (Krieger, 2011b, pp. 202–235) and Social-Ecological Systems theory (Box 1) in general (Ostrom, 2009), planetary health organizes multilevel determinants of human health within a system that explicitly accounts for biological, societal and environmental causes. Research focuses incorporate elements of human health, environmental health, and underlying socioeconomic contexts that influence the relationship between these different components (Cole & Bickersteth, 2018). Additionally, planetary health considers systematic feedback loops for causality – how human activities influence the health of natural systems and vice versa (Myers & Frumkin, 2020). By viewing societal impacts from both the human and natural systems perspectives, it becomes clear that human health is conditional on the health of natural systems that provide the underpinning ecosystem functions that support food production, clean water and air, and recreational space, among others (Corvalán, Briggs, & Zielhuis, 2000; Horton et al., 2014; “The Natural Capital Project”, 2020; Turner and Daily, 2008).

The academic field of planetary health emerged in response to ongoing environmental crises with the release of the Lancet-Rockefeller Commission Report in 2015 (Whitmee et al., 2015). The seeds of this field were planted by medical ecologists decades earlier (Dunk & Anderson, 2020; Nelson, Prescott, Logan, & Bland, 2019; Prescott & Logan, 2019), and the key idea – that the health of humans and their natural environments are interlinked – was held by indigenous cultures for millennia (Berry, 1991; Prescott & Logan, 2019). However, the emergence of planetary health as an academic endeavour serves as an umbrella to rally scientists and policymakers around a common problem, and shape research agendas fostered through multidisciplinary collaborations (Horton et al., 2014). This explicit effort to adopt perspectives across scientific domains allows for broader buy-in from scientists across fields ranging from ecology to earth sciences. Practitioners explicitly acknowledge the need for understanding how social systems can promote or harm planetary health, bringing in contributions from social scientists (Cole & Bickersteth, 2018; Myers, 2017). Planetary health encourages thorough examination of the interplay between human economic activities and natural systems changes. Problems that appear intractable from one scientific discipline's perspective might seem solvable by another. Evidence supporting a specific policy compiled across multiple fields of study provide more compelling support for that action.

Environmental scientists, physicians, and public health practitioners within the planetary health community have documented failures in humanity's stewardship of natural systems, leading to declining quality of ecosystem services (referring to benefits to humans provided by healthy ecosystems) worldwide (Myers & Frumkin, 2020; Myers, 2017). Steffen and colleagues reported dramatic covarying trends in human populations and economic activities alongside changes in earth systems which they called the “Great Acceleration” (Steffen et al., 2004) (Box 1). Concerns that human activities were harming earth systems led to the proposal of a Planetary Boundaries framework (Rockström et al., 2009) to define thresholds for environmental degradation (Box 1). The Planetary Boundaries concept provided a clear message that exceeding these thresholds of environmental degradation would have severe consequences for human health and the health of natural systems. The Intergovernmental Science-Policy Platform on Biodiversity and Ecosystem Services (IPBES) concluded in 2019 that ecosystem declines have accelerated in recent decades, with 47% declines in ecosystem extent and condition, 82% declines in biomass and species abundance, and 25% of species threatened with extinction (Diaz et al., 2019). Specific examples of human-led activities that exert unsustainable pressure on natural systems include climate change, stratospheric ozone depletion, land use change (deforestation, desertification, wetland loss), biodiversity loss, freshwater depletion, urbanization, and damage to coastal reefs and ocean ecosystems.

Planetary health scientists proceeded to characterize long-term risks to humans from the degradation of natural systems and identify opportunities to mitigate adverse health impacts, drawing from public health and epidemiologic research (Myers & Frumkin, 2020). Macro-level disruptions to natural systems exert human health effects through direct and indirect pathways. For example, direct health pathways include increased floods, heat waves, and pollution exposure, while indirect pathways include loss of livelihoods and population displacement (McMichael et al, 1999, 2006; Whitmee et al., 2015).

Planetary health research has identified key adverse environmental impacts of human activities that could have long-term consequences on human health. However, some of these natural systems alterations, such as land use change and urbanization, have historically been justified as a means to achieve short-term, local improvements in human health through economic development, despite risks to natural systems (Dinda, 2004). This poses challenges in achieving global targets like the Sustainable Development Goals which require economic growth for improved health and social welfare, but may require changing energy consumption and nutritional patterns to achieve environmental targets (United Nations, 2015). In the next section we review the development of the systems-oriented approach adopted by planetary health to relate human development, environmental degradation, and human health.

## 2 Historical context and conceptual frameworks for planetary health

### 2.1 The Kuznet's curve model of economic development, human health, and environmental degradation

**Fig. 1:**
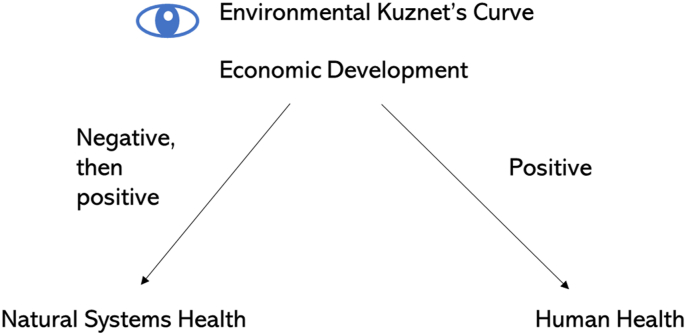
Environmental Kuznet's Curve (Dinda, 2004) represented by a simple conceptual model. Taking the perspective of Kuznet, Economic development leads to positive effects on human health, and initially negative but then positive effects on natural systems health.

Historically, the Environmental Kuznet's Curve was the dominant theoretical model explaining the relationship between economic development and environmental degradation. This model assumed that as incomes rise, so too would environmental degradation; however, once incomes grew sufficiently, people would advocate for greater environmental regulation and therefore limit further damage to the environment (Dinda, 2004). In more recent analysis leveraging large databases on the global distribution of risk factors and causes of death at different geographic scales and spaces, a more complex picture has emerged (Arrow et al., 1995; Smith and Ezzati, 2005). We can illustrate these relationships using a simple figure, in which the eye icon represents the perspective being taken (Fig. 1).

Economic development and environmental degradation in our globalized world are processes that unfold at different spatial and temporal scales. Environmental degradation is often displaced along spatial scales (local vs global) and temporal scales (short-term vs long-term impacts), and so beneficiaries of economic development may either not recognize these adverse health effects (McMichael, 2017; Smith and Ezzati, 2005), or see them as a price worth paying for development (Atapattu, 2002). For example, high-income countries have fewer household-level environmental concerns – such as indoor air pollution and lack of clean drinking water – as they began to industrialize. These household concerns are now more commonly found in low- and middle-income countries (Murray et al., 2020). High-income countries instead are concerned with reducing community-level risks, such as outdoor air pollution, occupational risks, and road traffic accidents (Smith and Ezzati, 2005). As countries accumulate sufficient wealth, these community-level risk factors decrease. However, global-level risks, such as contributions to climate change, exhibit a positive linear relationship with wealth, where the highest emissions are produced by high-income countries (O’Neill, Fanning, Lamb, & Steinberger, 2018; Smith and Ezzati, 2005). Within countries, environmental degradation often disproportionately burdens the poor, and minoritized racial and ethnic populations. Studies from the United States have reported that neighborhoods with larger non-White populations exhibit higher levels of black carbon pollution (Krieger, Waterman, Gryparis, & Coull, 2015), greater proximity to waste sites (Morello-Frosch Rachel, Manuel, Carlos, & James, 2002), and lower access to green spaces (Casey, James, Cushing, Jesdale, & Morello-Frosch, 2017). Most of the world's neglected tropical diseases occur among poorer populations in wealthy countries, including water-borne illnesses associated with poor sanitation (Hotez, Damania, & Naghavi, 2016), further illustrating within-country socioeconomic inequities.

This geographical displacement of environmental degradation distorts the relationship presented in the Environmental Kuznet's Curve, making it appear that environmental degradation is slowing in high-income countries at a more local scale, when in fact environmental degradation has been accelerating at a global scale. Furthermore, there is a temporal lag between the time at which environmental degradation begins and when the adverse health impacts can be observed. Thus, cross-sectional analyses of economic development and environmental degradation may discount the adverse impacts on human health. Without consideration of the entire global economic system and different spatiotemporal scales in which it operates, we may be misled by the apparent relationships between economic development and environmental degradation. This has led some to conclude that “we have been mortgaging the health of future generations to realize economic and development gains in the present (Whitmee et al., 2015).” Empirical examination of responsibility, capability, and vulnerability to climate change have shown that while high-income countries are more likely to be responsible for climate change and capable of mitigating harms, low- and middle-income countries are less responsible and more vulnerable to adverse health and economic effects (Füssel, 2010; Moore, Smith, Humphries, Dubrow, & Myers, 2020). This evidence can inform ongoing debates about politically tractable means for global distributive justice (Blake, 2012; Buse, Smith, & Silva, 2019).

### 2.2 Aligning human development goals with protecting the environment

Prior to the advent of planetary health, multidisciplinary teams of scientists had considered unified theories to explain the relationships between living and non-living organisms, and how they contributed to a healthy planet. Lovelock and Margulis’ Gaia theory proposed a symbiotic, interconnected relationship between living organisms and inorganic matter, that together optimized Earth for sustaining conditions for life (Lovelock, 2000; Lovelock & Margulis, 1974). Their crucial insight was that in nature, relationships between living organisms, their waste products, and inorganic matter operate through feedback loops that can protect the system as a whole from external shocks, analogous to the physiological concept of homeostasis. Later Social-Ecological Systems models (Berkes & Folke, 1994; Ostrom, 2009) explicitly incorporated sociopolitical factors to account for how human societies could either help or harm ecosystems adaptation and transformation, allowing ecosystems to either become more or less resilient to external shocks (Holling, 1973; Walker et al., 2004). These developments largely arose from complexity science, ecology, engineering, and economics, with a focus on general theories that could be explained using mathematical models capable of describing non-linear relationships and causal feedback (Gunderson & Holling, 2002).

In parallel, the fields of medicine and public health, building on long traditions of studying how places and environmental exposures led to human disease, began to recognize threats to human health posed by complex social and environmental phenomena (Krieger, 1994; McMichael, 1999; Susser and Susser, 1996). Environmental activism and growing concerns about the costs of environmental degradation required to support economic growth led some environmental health scientists to approach interconnections between humans and nature from a health-oriented lens (Dunk & Anderson, 2020).

### 2.3 Systems thinking frameworks to explain transformations of human and natural systems

Alongside these novel conceptual frameworks, ecology and economics researchers began incorporating systems thinking principles to model the complex, multilevel, dynamic relationships between human economic activities and natural systems.

The concept of panarchy proposed by Holling proposes that changes in human and natural systems arise through cycles of four distinct state transitions (“reorganization”, “conservation”, “creative destruction”, and “growth”) (Gunderson & Holling, 2002). When shocks are small, the system is resilient and remains in equilibrium. Large scale shocks, which are more likely during the “creative destruction” phase that is characterized by high unpredictability but low resilience, may prompt transformation of the system into a new one with a different equilibrium. The counterintuitive implication of the panarchy model is that, by exerting tight control over natural and political systems to behave in predictable ways in the short term, human activities may lessen resilience of those systems to adapt in face of rare but large stressors over the longer term (Holling, 1973). The severity of human and socioeconomic impacts of the SARS-COV-2 pandemic, a rare but intense external shock, have illustrated how fragile our tightly controlled economic and health systems are.

Another systems model for explaining organization of human and natural systems is the Social-Ecological Systems model, proposed by Ostrom (Ostrom, 2009). The model was initially proposed to guide policies for managing of environmental resources which generally sit outside traditional governance structures. The model assumes that local actors influence one another at the micro-level, leading to emergence of changes at macro level. The model also defines relationships between all actors (human and non-human) as networks, in which any component of the network can exert some influence on the overall system. Under this conceptualization, humans are a part of, rather than separate from, nature. Within the Social-Ecological Systems model, “adaptation” refers to the systems' ability to draw from experiences and knowledge contained by its actors and adjust response to maintain stability. “Transformation” refers to the system's capacity to create a new system when ecological, economic, or social shocks are too extreme to be managed by its actors.

Examples of systems thinking models applied in public health include assessments by international agencies to assess relationships between economic indicators, natural resource availability, and socioeconomic indicators (Probst & Bassi, 2017). The United States Environmental Protection Agency has employed systems-based modelling to understand challenges with managing land use, coastal pollution, and social wellbeing (Pongsiri, Gatzweiler, Bassi, Haines, & Demassieux, 2017). Practitioners emphasize the need to understand local context and how policy changes may lead to unintended impacts at different spatiotemporal scales.

### 2.4 The doughnut: a framework for sustainable human development within the bounds of social justice and planetary health

**Fig. 2:**
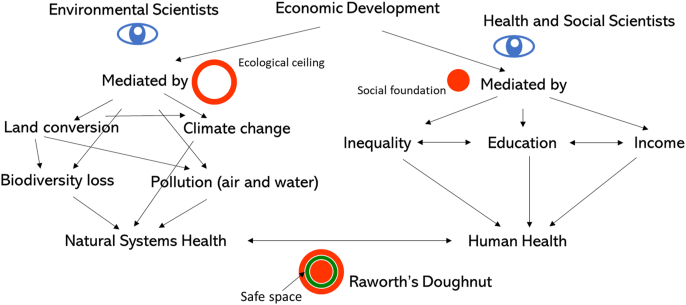
Raworth's doughnut (Raworth, 2017) represented by a conceptual model. The model takes perspectives of climate scientists, health and social scientists, and planetary health (represented by the eyeball icon). The model simplifies the pathways through which economic development influences human health and the health of natural systems. From health and social sciences, we see that components of the social foundation, including inequality, education, and income, influence human health. From climate science, we see that economic development leads to pressures on natural systems health through land conversion, climate change, biodiversity loss, and pollution. Planetary health and the doughnut framework allow us to connect these impacts of economic development on natural systems health and human health in far more complex ways than the Kuznet's curve.

In 2012, Raworth proposed a systems framework for guiding sustainable human development called the “doughnut model” (Fig. 2) (Box 1). In the doughnut model (Raworth, 2017), policies promoting human development must safely and justly operate within the space contained by the outer and inner circles that define the doughnut. Human activities that fail to consider social and political factors exert pressure on the inner boundary of the doughnut, or the “social foundation.” The twelve dimensions that comprise the “social foundation” were derived from the United Nations Sustainable Development Goals: water, food, health, education, housing, income and work, peace and justice, political voice, social equity, gender equity, networks, and energy (Raworth, 2017). Human development also exerts pressure on the outer layer of the doughnut, or the “ecological ceiling”, which encompasses planetary thresholds on supporting life. The Planetary Boundaries framework includes nine Earth Systems dimensions impacted by humans (Rockström et al., 2009). The doughnut model incorporates perspectives of human health and the health of natural systems through the concepts of social foundation and ecological ceiling, and reveals how boundary components are interlinked within a broader planetary health system. Although the doughnut has been criticized for lacking guidance on how to prioritize different elements of the social foundation and ecological ceiling or consider trade-offs (Evison & Bickersteth, 2020), it remains a useful tool for guiding societal values and thinking through unintended environmental, health, and social consequences of different policy decisions.

**Fig. 3:**
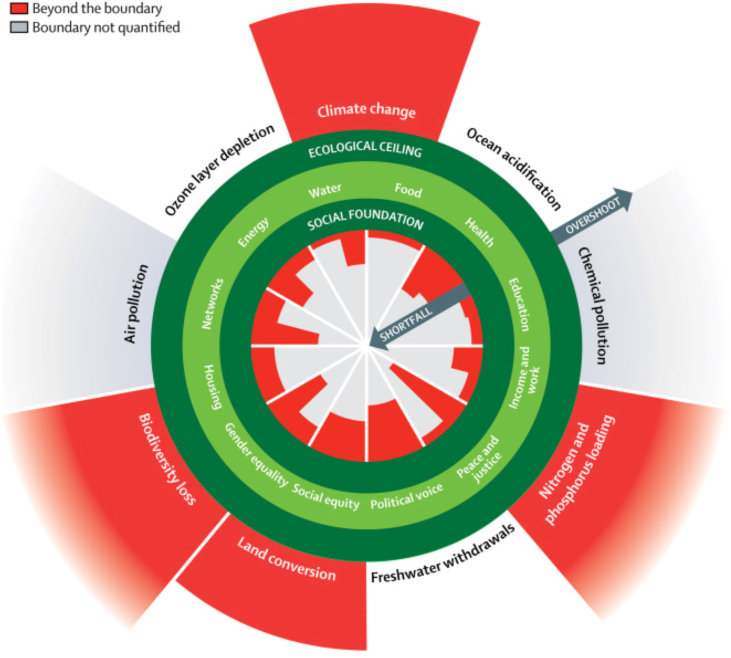
Global shortfalls in the social foundation and overshoot of the ecological ceiling in the Doughnut. Source: Raworth K, A Doughnut for the Anthropocene: humanity's compass in the 21st century. The Lancet Planetary Health 2017; 1e48-e49. https://doi.org/10.1016/S254205196(1730028-1). Reproduced under the Creative Commons, license CC BY 4.0 (Raworth, 2017).

A recent analysis applying the doughnut framework to study progress towards global Sustainable Development Goals using indicators collected between 1995 and 2016 found threats to both the social foundation and ecologic ceiling (Fig. 3). Using metrics provided by international bodies including the United Nations, World Health Organization, and World Bank, the authors found evidence of gaps in attainment of gender equity (using measures of female parliamentary representation and earnings gap), social equity (using measures of income inequality), political voice (using the World Bank Voice and Accountability Index), and peace and justice (based on corruption and homicide rate per 10,000). In addition, the report found evidence of overshooting the ecological ceiling in climate change (169% of the threshold of a 350 parts per million concentration of atmospheric carbon dioxide), biogeochemical cycles (229% over the threshold of 6.2 million tons of phosphorous and 62 million tons of reactive nitrogen applied to land fertilizer per year), land conversion (62% of area of forested land as a proportion of forest-covered land prior to human alteration, compared to ideal of 75%), and biodiversity loss (a rate of 100–1000 species extinction per million species per year compared to a threshold of 10 or less per year). O'Neill et al. conducted a global cross-country analysis to study whether countries with strong social foundations were more or less likely to protect planetary boundaries. In a global country-level analysis, they observed a strong positive correlation between the number of social thresholds achieved and biophysical boundaries transgressed, with higher income countries more likely to achieve high social development but also high planetary boundaries transgressions (O'Neill et al., 2018). A challenge for modern societies is how, once social goals are achieved, to avoid transgressing the planetary boundaries within the doughnut.

## 3 Using the doughnut and systems thinking to identify human and natural systems co-benefits to sustain planetary health

The doughnut framework provides a useful tool for encouraging cross-sectoral policy research to promote planetary health. The doughnut can be easily explained to stakeholders within different policy domains, as well as different actors in society such as those in business, local communities, and government. Public health practitioners across different disciplines have advocated for their peers to leverage their professional bodies to change industrial practices, serve as community role models, and advocate for policies that sustain planetary health (Box 2). The doughnut could therefore serve as a common reference point enabling these diverse stakeholders to prioritize actions to protect the social foundation and ecological ceiling. Furthermore, the doughnut offers a framework for identifying sectors of the economy that apply undue pressure on social and ecological boundaries (Brannigan, 2020; Olsson, 2020; Raworth, 2017). Two recent examples leveraging research from climate science and public health have been offered by planetary health scientists – the global food system and rapid urbanization.

Box 2 **Calls by Health Professionals to Commit to Planetary Health through their training, work and advocacy**

**Table:** 

Given its longstanding commitments reducing morbidity and mortality and achieving social justice, public health is well-placed to act at individual, societal, and global level to protect planetary health. All allied health professionals should share these professional goals (Horton et al., 2014; Wabnitz et al., 2020) General practitioners and primary care physicians should implement planetary health practices through ensuring that their businesses employ green and sustainable practices, and advocating for behaviors and lifestyles among their patients and communities that promote planetary health. At individual level, pursue lifestyles that limit climate impact, encourage networks to do so, and support political causes that align with planetary health. As a physician, counsel patients to prepare for climate change, adopt lifestyles that mitigate their own impact. As a professional body, state a unified position on climate change and planetary health and advocate for policies that mitigate adverse impacts. (Kemple, 2019; Pendrey, Beaton, & Kneebone, 2020; Xie et al., 2018) Physicians should include planetary health principles as part of education. Calls for reducing impact of health care industry through decarbonization. (Silverman, 2019; Veidis et al., 2019) Nurses should adopt a social justice lens and embrace planetary health principles to better align professional goals and objectives with addressing social determinants of health. (Rosa and Upvall, 2019) Public health education should include courses that prepare trainees for addressing climate change. (Silverman, 2019) Allied health professionals should pledge their commitment to planetary health, analogous to pledges of the Hippocratic Oath taken by physicians (Wabnitz et al., 2020) Global surgery argues that climate change will lead to greater demand for surgical services. There is a need for the profession to consider how climate disasters will impact ability to deliver high quality surgical care. Surgical programs should adopt sustainability practices in delivering care by reusing equipment when possible, advocate for planetary health policies, support research on sustainable surgical practice, and advocate for reducing emissions in the health care system (Roa et al., 2020)

Alt-text: Box 2

### 3.1 Systems-based conceptual framework for effects of the global food system on health of humans and natural systems

Today's global food systems is failing to provide nutritious diets for humanity or to preserve the global environment. Worldwide, 820 million people don't have enough to eat, a number that has been steadily growing since 2015 after a decade of decline (FAO, IFAD, UNICEF, WFP and WHO, 2020). Two billion people globally are estimated to be experiencing micronutrient deficiencies, predominantly in iron (Stoltzfus, 2003; Zimmermann and Hurrell, 2007) zinc (Brown, Wuehler, & Peerson, 2001; Caulfield & Black, 2004; Wessells and Brown, 2012), vitamin A (*Global Prevalence of Vitamin A Deficiency in Populations at Risk 1995–2005**: WHO Global Database on Vitamin A Deficiency*, 2009; Stevens et al., 2015), and protein (de Onís, Monteiro, Akré, & Glugston, 1993; Medek, Schwartz, & Myers, 2017). Meanwhile, populations in many countries are also facing a pandemic of obesity and metabolic diseases from excess caloric and salt intake, with over 2 billion adults worldwide overweight and obese (Micha et al., 2020, p. 2020; NCD Risk Factor Collaboration (NCD-RisC) (2016). Inadequate intake of healthy foods such as fruits, vegetables, and nuts is also driving large burdens of disease (Stanaway et al., 2018). In light of these persistent challenges, strategies for global food and nutritional security have begun to shift from strictly producing adequate calories to providing more nutritious diets (FAO, 2020; Global Panel on Agriculture and Food Systems for Nutrition, 2020; Willett et al., 2019).

Coincident with the recognition that more nutritious diets are required is growing awareness that global food production, primarily through agricultural practices, exerts pressures across domains of the ecological ceiling of the doughnut (Godfray & Garnett, 2014; Myers, 2020; Willett et al., 2019). Agriculture is a significant contributor to climate change, responsible for between 25 and 33% of global greenhouse gas emissions (Mbow et al., 2019). From 1961 to 2010, fertilizer and pesticide consumption increased for 35–40% of countries around the world, with the mean annual consumption of top 10 countries ranging from 1.89 million tons (Canada) to 21.6 million tons (China) (Liu, Pan, & Li, 2015). Increases in nitrogen and phosphorus from soil erosion have become key drivers of ecosystem change (Rockström et al., 2009). Excess nitrogen running into land ecosystems leads to decreased plant diversity, and high amounts of nitrogen and phosphorus in bodies of water lead to harmful algal blooms and depleted levels of dissolved oxygen in inland waters and coastal areas (Hales, McMichael, & Butler, 2005).

Agricultural activities have also led to dramatic global changes in land use (Myers, 2020). Deforestation from agricultural activities has accelerated in recent years, especially in tropical regions (Hosonuma et al., 2012; Mayaux et al., 2013; Whitmee et al., 2015). Though the rate of deforestation occurs predominantly in low- and middle-income countries, demand for products of that deforestation, such as palm oil and soya bean monocrops, are global, highlighting how consequences of economic actions are displaced (Whitmee et al., 2015). Deforestation leads to loss of biodiversity as habitat shrinkage and simplification reduces wildlife population numbers, and drives some species to extinction (Barnosky et al., 2011). Deforestation may also lead to changes in local climate and temperature, and the release of carbon from soil and vegetation (Myers, 2020; Tinker et al., 1996). Soil degradation also has accelerated desertification, reducing arable land (Olsson et al., 2019). Desertification may result in adverse changes to planetary systems that reduce global heating. For example, desertification is associated with loss of atmospheric albedo that reflects solar radiation, leading to greater warming.

The global food system also exerts pressures on the social foundation of the doughnut. Agriculture-driven deforestation and land use changes adversely affect human health through emerging outbreaks of infectious diseases. For example, pathogens such as Human Immunodeficiency Virus (Sharp and Hahn, 2011) and Ebola (Groseth, Feldmann, & Strong, 2007) emerged through novel human-wildlife interactions. The burden of other infectious diseases, including malaria (Santos and Almeida, 2018) have also increased following rapid deforestation, as insect vectors such as mosquitoes move to previously uninhabitable areas (Myers, 2017; Myers et al., 2013). In addition, deforestation can foster spread of waterborne infectious diseases (Ellwanger et al., 2020; Herrera et al., 2017) and exert direct impacts on food security and nutrition (Hickey, Pouliot, Smith-Hall, Wunder, & Nielsen, 2016). Westernization of diets characteristic of the nutrition transition are rich in red meat, salt, and sugar-sweetened beverages, and highly processed foods, all of which contribute to heart disease, diabetes, and some cancers (Willett et al., 2019; Wong et al., 2017). Increases in beef production contribute to deforestation (Nepstad et al., 2014) and greenhouse gas emissions (Lynch, 2019; Reisinger & Clark, 2018). The accompanying soil runoff introduces high levels of nitrogen and phosphorus pollution in freshwater sources that can spread pathogens, thereby reducing global availability of clean freshwater (Hales et al., 2005). Construction of dams to address food shortages can also perturb local ecosystems leading to outbreaks of waterborne diseases such as schistosomiasis (Shaikh et al., 2018). Climate change can then push farmers towards less water-intensive but ultimately more damaging practices, such as from crop farming to poultry rearing, risking feedback loops that further threaten to put pressure on already stressed systems (Cole & Desphande, 2019).

Planetary health scientists have undertaken modelling studies to determine policies that could offer co-benefits to human health and health of natural systems through changes in diet practices and changes in the global food system. A recent analysis investigated projected environmental impacts arising from increasing population growth and subsequent food demand, assuming trends in food production would increase based on 2010 levels (Springmann, Clark, et al., 2018). This analysis revealed that environmental pressures on greenhouse gas emissions, cropland use, bluewater use, nitrogen application, and phosphorus application would increase by 50–92% if no technological advances were made. Reducing food loss and waste, improving efficiency of crop and food production through technology improvements, and changing diets could lead to as much as 30–60% reductions in environmental impacts. A related analysis compared impacts of adopting three different diets (replacing animal-with plant-based foods, reducing levels of underweight and overweight people, and adopting energy-balanced flexitarian, pescatarian, vegetarian, and vegan diets) on human health and planetary boundaries (Springmann, Wiebe, et al., 2018). Models suggested that adopting energy-balanced low-meat diets led to premature mortality reductions ranging from 19% to 22%, as well as reduced environmental impacts (54–87% lower greenhouse gas emissions, 23–25% lower nitrogen application, and 18–21% lower phosphorus use). However, they noted that there was geographic variation in distribution of benefits, with higher income countries benefitting more than low-income countries. While there is growing consensus for the need for more efficient food systems and adoption of more nutritious, environmentally friendly diets, some have argued that global studies may mask important local variations in cultural acceptability of diets, health, environmental, and societal priorities (Béné et al., 2019). These critics advocate for multidisciplinary discussions about how to define sustainability and adoption of systems-based conceptual frameworks that account for multilevel hierarchies of actors within the food system, and feedback between changes in food systems and diet practices.

**Fig. 4:**
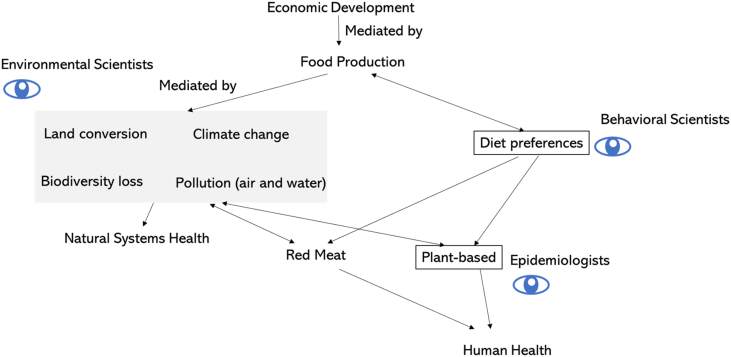
Conceptual model for identifying modifiable societal behaviours that influence adverse health and natural systems impacts of the global food system. Economic development influences food production, which is influenced by diet preferences. Climate science shows how the food system influences adverse effects on natural systems through land conversion, climate change, biodiversity loss, and pollution. Behavioural science and epidemiology show how diet preferences lead to consumption of red meat and plant-based diets, which exert different effects on human health. Modifying diet preferences to switch consumption from red meat to plant-based diets would lead to improved human and natural systems health. (For interpretation of the references to colour in this figure legend, the reader is referred to the Web version of this article.)

Planetary health scientists apply systems thinking principles to bridge knowledge from environmental sciences and public health. This intersectoral collaboration has identified co-benefits to humans and natural systems that would arise from targeted policies to adopt sustainable food production practices, influence dietary choices, and reduce food waste (Myers, 2020). These changes would be accompanied by human health benefits including lower obesity, cardiometabolic disease, and mortality (Abete, Romaguera, Vieira, Lopez de Munain, & Norat, 2014; Orlich et al., 2013; Stanaway et al., 2018), as well as natural systems health benefits including lower land conversion and pollution (Drew, Cleghorn, Macmillan, & Mizdrak, 2020; Springmann, Clark, et al., 2018) and arresting climate change and biodiversity loss. These relationships are summarized in Fig. 4. While much work remains to bring these policies to fruition, applying the doughnut to the global food system and its impacts on human health and the health of natural systems helps identify important policy leverage points.

### 3.2 Systems-based conceptual framework for understanding how urbanization affects human health and the health of natural systems

Over half of the world's population now lives in towns and cities and an estimated 2.5 billion people, or 68% of the world's population will live in urban areas in 2050 (United Nations, Department of Economic and Social Affairs, Population Division, 2018). Land use changes associated with urbanization influence human health through changing lifestyles and behaviors, particularly in low- and middle-income countries (Aznar-Sánchez, Piquer-Rodríguez, Velasco-Muñoz, & Manzano-Agugliaro, 2019; Diez Roux et al., 2020; Ezeh, 2016; Vlahov and Galea, 2002). Land use and transportation systems in cities shape human health behaviors through connectivity of streets and access to transportation systems (Sallis et al., 2012). Features of cities, such as walkable streets and access to parks, affect routine physical activity by providing opportunities to conduct daily activities using active transportation (i.e., walking, bicycling) as opposed to driving. Transport systems influence per capita emissions of air, water, light, and noise pollution (Giles-Corti et al., 2016; Glazener & Khreis, 2019; Nieuwenhuijsen & Khreis, 2016). Urban design can therefore have profound downstream consequences for human health.

Although cities offer many health, economic, and social benefits, unplanned urban growth and sprawl exert pressures on the ecological ceiling and the social foundations of the doughnut (Diez Roux et al., 2020; Galea, Freudenberg, & Vlahov, 2005). Urban areas with less vegetation and more impervious surfaces (such as asphalt and concrete) can concentrate heat without restorative cooling abilities, resulting in a “heat island” effect (Bonan, 2008; Chakraborty, Hsu, Manya, & Sheriff, 2020) that is linked directly to negative health outcomes (Heaviside, Vardoulakis, & Cai, 2016) and may exacerbate cardiovascular mortality from air pollution as well as the impact of heat stress alone (Analitis et al., 2018), and pollen exposure leading to increased allergies (Anenberg, Haines, Wang, Nassikas, & Kinney, 2020). Heat islands also increase energy demands for cooling and related greenhouse gas emissions (US EPA, 2014).

However, properly designed cities can lessen these pressures. Health and social scientists have shown that cities exert positive pressure on the social foundation by promoting social equity, gender equity, political voice, income and work, education, health, and networks by concentrating high income, socially productive citizens. Cities may also reduce per capita resource use and pollution, or urban dwellers’ “ecological footprints” relative to rural dwellers (Meyer, 2013). Low population density urban design may exacerbate pollution by increasing traffic. Transportation generates approximately 29% of greenhouse gas emissions in the United States, the largest of any individual sector (US EPA, 2015). These examples illustrate how appropriately managing land use change that accompanies urbanization could lead to co-benefits for human health and the health of natural systems (Geist & Lambin, 2002; Lambin & Meyfroidt, 2011).

Epidemiologic evidence supports the hypothesis that exposure to nature in urban areas leads to greater human health (Gascon et al., 2016; Lee & Maheswaran, 2011). These benefits include improved mental health, cognition, lower stress, higher physical activity, lower incidence of cardiovascular disease and certain cancers, higher birth weights, and lower mortality rates (Bratman et al., 2019; Demoury et al., 2017; Fong, Hart, & James, 2018; Frumkin, 2013; Frumkin et al., 2017; Iyer et al., 2020; James, Banay, Hart, & Laden, 2015; Jimenez et al., 2021; Markevych et al., 2017). Exposure to nature contact may also produce more favorable immune profiles (Hanski et al., 2012), following the “hygiene hypothesis” positing that early exposure to diverse microbes may protect against allergies (Strachan, 2000). However, some studies find that increased nature exposure is associated with higher rates of asthma and allergens, particularly in children (Dadvand et al., 2014; Ferrante et al., 2020; Fuertes et al., 2014; Jimenez et al., 2021). Although the strength of evidence is still evolving, this research suggests that incorporating natural features into urban environments might have benefits for human health (Shanahan et al., 2015). Incorporating green spaces into the design of high-rise apartments could offset mental health disparities reported by residents in low-income neighborhoods (Larcombe, van Etten, Logan, Prescott, & Horwitz, 2019).

**Fig. 5:**
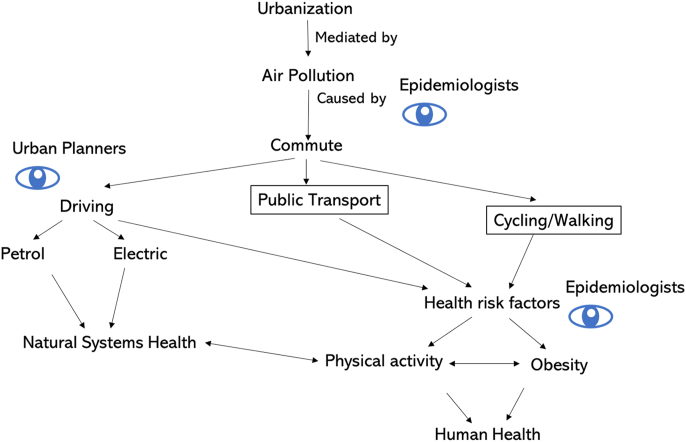
Conceptual model for identifying modifiable societal behaviours that influence adverse health and natural systems impacts of urbanization. Urbanization leads to air pollution that is influenced by commuting patterns of residents. Public health scientists and urban planners can study different types of transport, and effects of incentivizing use of public transport and cycling/walking, which lead to better human health through higher physical activity and lower obesity. Reducing reliance on driving also leads to better natural systems health through lower emissions.

**Fig. 6:**
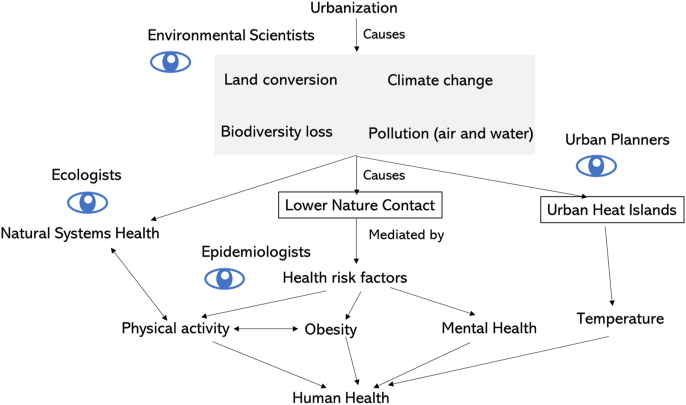
Conceptual model for identifying modifiable aspects of urban form that lead to adverse health of humans and natural systems. Unplanned urbanization contributes to land conversion, climate change, biodiversity loss, and pollution, leading to poorer natural systems health. These changes in natural systems impact human behaviour through lower nature contact and the presence of urban heat islands. Lower nature contact is associated with numerous health risk factors (physical activity, obesity, mental health) and other chronic disease and mortality outcomes in humans. Temperature is associated with increased risk of mortality at lower and upper extremes.

Planetary health scientists have begun to compile evidence of co-benefits that would arise from more thoughtful urban planning (Fig. 5, Fig. 6). Numerous studies in Europe are beginning to model impacts of changing urban transport patterns by switching car trips to public transport, cycling or walking (Mueller et al., 2015; Rojas-Rueda et al., 2012). These studies suggest that population-level adoption of these policies could lead to appreciable mortality reductions and lower carbon emissions (Rojas-Rueda et al., 2012). In addition, increasing nature contact, either through promoting travel to and use of green spaces or by introducing more green vegetation to urban areas, could improve mental and physical health as described above (Fig. 6). Growing evidence suggests that increasing access to green spaces and parks, particularly among socially disadvantaged populations, could lead to reductions in health inequities (Mitchell, Richardson, Shortt, & Pearce, 2015; Rigolon et al., 2021), but interventions should consider local context, the safety of the spaces, and community beneficiaries’ practices towards green spaces (Shanahan et al., 2015). Forests and trees serve as important carbon sinks and can lower urban temperatures, thereby reducing adverse impacts of urban heat islands.

Cities also are where the starkest inequalities in income, education, and health can be found (Abel & Dietz, 2019). In many cities, legacies of historic discrimination based on race and social class are geographically patterned (Casey, James, et al., 2017; Krieger, 2013; Nardone, Casey, Morello-Frosch et al., 2020; Nardone, Casey, Rudolph, et al., 2020; Nardone Anthony et al., 2021). The geographic distribution of income, wealth, and education in cities reveals that people of lower socioeconomic position are generally found in neighborhoods with fewer professional opportunities, higher crime rates, and nearer to sources of environmental pollution (Mohai, Pellow, & Roberts, 2009). These neighborhoods may also have less access to health care services, leading to poorer outcomes (Nardone et al., 2020b, Nardone et al., 2020a). Other contextual environmental factors, including green space and noise exposures, have been shown to favor high-income White populations relative to others in the US (Casey, James, et al., 2017; Casey, Morello-Frosch et al., 2017). Studying land use patterns in cities and their relationship with health equity could reveal policy changes that will reduce disparities between more and less privileged members of society, thereby improving the social foundation of the doughnut.

### 3.3 Evaluating impacts of hypothetical policies on human health and health of natural systems

Public health scientists and practitioners have numerous tools to apply systems thinking principles to sustain planetary health. For example, the Driving forces, Pressures, State, Exposures, Effects and Actions (DPSEEA) framework has been used by the WHO and national governments in the Americas to understand how human activities expert pressure on environments, and how changing environmental states lead to adverse human health impacts through changes in exposures (Carneiro Fernando et al., 2006; Corvalán et al., 2000; Gentry-Shields & Bartram, 2014). This framework was designed to leverage existing information systems, thereby avoiding costs of additional data collection. The WHO European Region piloted a DPSEEA-derived model called the Environment and Health Information System (EHIS) to determine the causal chain between driving forces of environmental degradation and downstream health consequences to determine where action could be taken to mitigate harms (WHO Europe, 2010). Similarly the Health and Environment Linkages Initiative (HELI) sponsored by the WHO and United Nations Environmental Programme (UNEP) adopted the DPSEEA framework to support Low- and Middle-Income Countries with acquiring data on environmental equality and implementing economic evaluations to inform policies to control adverse health impacts of environmental degradation related to water management and agricultural practices (The WHO-UNEP Health and Environment Linkages Initative (HELI), 2004). In addition, the doughnut framework could be used by scientists and policymakers to understand unintended environmental and social outcomes of various policies and described barriers to adopting these frameworks.

Analytic approaches to evaluate impacts of human behaviors on the environment include the Health Impact Assessment (HIA) (Osofsky & Pongsiri, 2018) in coordination with Environmental Impact Assessment (EIA). EIA was established in the 1970s to evaluate the likely environmental impacts of a proposed policy accounting for beneficial or harmful socioeconomic, cultural, and less consistently, human health impacts (US EPA, 2013). In parallel, HIA is an approach for evidence-based planning or policy making with the direct aim of improving human health (Lock, 2000). HIA is a combination of methods to assess the health outcomes of a policy that does not include health as its primary focus. Combining the approaches of HIA and EIA to evaluate policies would allow policymakers to assess multilevel effects of policy recommendations, and ensure decisions are informed by both their potential human health and natural systems impacts (Osofsky & Pongsiri, 2018). Findings of HIA and EIA models are expected to involve uncertainty because their goal is to accurately predict future impacts of policy decisions. Therefore, HIA and EIA require robust sensitivity analysis and retrospective validation of models (Frumkin & McMichael, 2008). Studying the complex interactions between human development, environmental change, and human health at macro-level scales will require use of designs more common in econometrics than epidemiology, such as difference-in-difference (Angrist, 2008) and interrupted time series (Bernal, Cummins, & Gasparrini, 2017). Ultimately, iterative and integrative HIA and EIA can propel the field of planetary health through deeper understanding of dynamic relationships between human health and the environment.

Specific examples of HIA to assess co-benefits of human and natural systems health are already underway. Public health scientists have tested interventions to leverage co-benefits from encouraging switching from car use to active transport for commuting and navigating cities. Using data from mostly European countries, researchers have demonstrated that switching from motorized to active transport yields tremendous benefits to health accruing from increased physical activity (Mueller et al., 2015). In addition, a study in Barcelona suggested that switching 40% of round-trip travel to the city from cars to cycling would result in 66 fewer deaths from physical activity benefits, 10 fewer deaths due to reduction in air pollution, and a reduction of 203,251 tons of carbon dioxide per year (Rojas-Rueda et al., 2012).

Legal mandates for HIA and EIA can incentivize policymakers to consider both health and environmental impacts when making decisions. Different countries have embraced these appraisal methods when discussing business activities, policies to change land use mix, and transportation projects. However, countries differ in the extent to which HIA and EIA are jointly considered. For example, in European and Canadian settings, HIA is often considered together with EIA, but in the United States, EIA conducted at the federal level often does not include health impacts. A review of global implementation of HIA found that, though legal mandates increased systematic appraisals of both health and environmental consequences of policy proposals, long-term sustainability required additional resources. Specifically, the report recommended that agencies tasked with conducting HIA and EIA be provided additional training in technical skills, and resources for developing standards for rigorous assessment (National Research Council (US) Committee on Health Impact Assessment, 2011, pp. 130–176). Nonetheless, legal mandates for HIA and EIA could provide incentives for the doughnut and similar models to be systematically considered when proposing public health and other policies.

Public health professionals would be well-placed to conduct these appraisals and advocate for policies that protect planetary health. Advancing the use of HIA and EIA requires careful consideration of specific policies to compare, which health and environmental metrics to study, and communication of evidence-based recommendations to decision-makers and communities. Following the systems-oriented principles adopted by planetary health, public health practitioners should engage stakeholders in non-health sectors, such as urban planning, agriculture, ecology, and economics, when designing HIA and EIA proposals (Fig. 6). In addition, public health practitioners should leverage their contextual knowledge to advocate on behalf of the communities they serve, as public health practitioners will often have both understanding of health care needs and trust of these communities. When conducting HIA and EIA, public health practitioners and researchers should, when appropriate, apply systems thinking conceptual models and analytic approaches that embrace dynamic feedback between variables, multilevel hierarchies, and uncertainty.

## 4 Conclusion

In summary, systems thinking is essential for considering how human activities influence human health and the health of natural systems. Taking a systems-wide approach when evaluating impacts of policies will encourage examination of unintended consequences of these actions at different spatial and temporal scales. Organizing knowledge regarding impacts of human activities on health and natural systems and incorporating multiple perspectives is key to ensuring the policies are recommended within the appropriate context.

While the scale of the problems that global environmental change poses to humans and our planet is immense, public health professionals are equipped with the skills, experience, and commitment to maintaining life support systems within planetary boundaries to allow humanity to thrive. As public health professionals, contributions from fields ranging from sociology to environmental science have enriched the knowledge base that public health draws from, uniquely allowing us to view the complex systems and draw from multiple scientific domains to inform policy. Our methodological expertise allows us to conduct rigorous research and communicate our findings to key decision-makers and the public. Lastly, our field's experience with advocacy for the most vulnerable and marginalized members of society ensures that we will support solutions that promote equity. As researchers, we have an obligation to continue pursuing inquiries at the intersection of climate science, human health, and policy to ensure that the best scientific evidence can be drawn from in service of human and ecosystem health. As professionals, we must use our trust and authority to take stands on these issues within our professional bodies as many health care provider associations have done (Box 2), and better understand the impact of our professions on the ecological ceiling and social foundation proposed by Raworth's doughnut. As individuals, we should consider the impacts of our social, political, and economic choices and how they will impact our health and the health of our descendants (Foster, Cole, Farlow, & Petrikova, 2019).

Since planetary health emerged as a field, public health has contributed to two major policy recommendations – adapting our food system to promote plant-based diets, and transforming our cities to reduce their environmental impact and improve the health of their residents – that can help mitigate humanity's impact on the planet while sustaining health gains made over the past century. These focal areas illustrate how public health professionals can support policies that operate within the safe space of Raworth's doughnut framework by considering impacts of policies on social wellbeing and ecosystem services. This will require us to rely on more complex analytic approaches, informed through interdisciplinary collaboration between environmental, health and behavioural scientists, that capture the dynamic relationships between human activities, human health, environmental change, and environmental degradation. Many more human and natural systems co-benefits remain to be identified. Public health professionals are well positioned to lead this effort and indeed, must do so to preserve the health and longevity of the public and our planet.

## Author contribution

**Hari S. Iyer,** Conceptualization, Writing – Original Draft, Writing – Review & Editing. **Nicole V. Deville,** Conceptualization, Writing – Review & Editing. **Peter James,** Conceptualization,. Funding acquisition, Supervision, Writing – Review & Editing. **Christopher D. Golden:** Conceptualization, Supervision, Writing – Review & Editing, **Olivia Stoddard,** Data Curation, Writing – Review & Editing. **Huichu Li,** Data Curation, Writing – Review & Editing. **Elise G. Elliott,** Data Curation, Writing – Review & Editing. **Marcia P. Jimenez**: Data Curation, Writing – Review & Editing. **Jennifer Cole,** Writing – Review & Editing. **Samuel S. Myers,** Writing – Review & Editing.

## Funding

HSI, NVD, and PJ were supported by the 10.13039/100000002National Institutes of Health (HSI: T32 CA 009001, NVD: NIH T32 ES007069, PJ R01 HL150119). CDG was supported by the 10.13039/100000001National Science Foundation (CNH 1826668). This research was funded by the 10.13039/100006363National Geographic Society.

## Financial declarations

The authors declare no competing interests.
